# Hydrotherapy in the Rehabilitation of Functional Performance and Gait in Knee Osteoarthritis: A Systematic Review of Randomized Controlled Trials

**DOI:** 10.3390/medicina62050994

**Published:** 2026-05-19

**Authors:** Mihaela Minea, Andreea-Alexandra Lupu, Andreea-Dalila Nedelcu, Viorela-Mihaela Ciortea, Laszlo Irsay, Mădălina-Gabriela Iliescu

**Affiliations:** 1 Faculty of Medicine, Doctoral School, “Ovidius” University of Constanta, 1 University Alley, Campus-Corp B, 900470 Constanța, Romania; mihaeala.minea@365.univ-ovidius.ro (M.M.); dalila.nedelcu@365.univ-ovidius.ro (A.-D.N.); 2 Hospital Rehabilitation Unit, Balneal Sanatorium of Techirghiol, Victor Climescu Street 34–40, 906100 Constanța, Romania; 3 Department of Medical Rehabilitation, Faculty of Medicine, “Ovidius” University of Constanța, 1 University Alley, Campus-Corp B, 900470 Constanța, Romania; 4 Department of Rehabilitation Medicine, University of Medicine and Pharmacy “Iuliu Hațieganu”, Victor Babeș Street 8, 400012 Cluj-Napoca, Romania; viorela.ciortea@umfcluj.ro (V.-M.C.); laszlo.irsay@umfcluj.ro (L.I.)

**Keywords:** knee osteoarthritis, hydrotherapy, functional performance, gait-related outcome

## Abstract

*Background and Objectives*: Knee osteoarthritis (KOA) is a degenerative joint disease that affects quality of life through pain, impaired functional performance, and altered gait patterns. Hydrotherapy is a well-tolerated form of physical rehabilitation, especially suitable for patients with severe pain, as water’s properties support movement while reducing joint load. Its effects have been widely studied, primarily focusing on patient-reported outcomes, with limited synthesis of functional performance and gait-related outcomes. *Materials and Methods*: A systematic search was conducted in PubMed, Web of Science, Cochrane, PEDro, SpringerLink, ScienceDirect, and Google Scholar, following the Preferred Reporting Items for Systematic Reviews and Meta-Analyses (PRISMA) guidelines. The search strategy included a combination of Medical Subject Headings (MeSH) terms and keywords. For example, the PubMed search strategy was as follows: (“knee osteoarthritis” OR “knee OA”) AND (“hydrotherapy” OR “aquatic therapy” OR “water-based exercise”) AND (“gait” OR “walking” OR “functional performance”). Randomized controlled trials (RCTs) from the last 10 years involving patients with KOA undergoing aquatic therapy were included. Primary outcomes included functional performance assessed by measures such as the 6 min walking test (6MWT), the Timed Up and Go (TUG) test, the five sit-to-stand (5 STS) and stair climb (SC) tests, and by using gait-related parameters (e.g., speed, cadence, and step length) assessed clinically or using technology. Patient-reported outcomes, including the Visual Analog Scale (VAS), Western Ontario and McMaster University’s Osteoarthritis Index (WOMAC), and Knee Injury and Osteoarthritis Outcome Score (KOOS), were analyzed as a secondary objective. *Results*: A total of 479 studies were identified, of which 13 met the eligibility criteria. The results revealed improvements in functional performance, with increases in 6MWT in five studies, the TUG test in four trials, and better performance in the 5-STS and SC tests in five studies. Benefits in gait parameters were noted in four studies. Additionally, one of the articles reported improvements in static and dynamic balance, another showed enhanced proprioception, and a third described more efficient muscle activation during gait following hydrotherapy. Consistent benefits in pain reduction, joint stiffness, and activities of daily living, as reflected by VAS, WOMAC, and KOOS, were also noted immediately and maintained at follow-up. The variability in outcome measures and intervention characteristics limited the possibility of data integration and the calculation of effect sizes. *Conclusions*: Hydrotherapy as a rehabilitation intervention may be associated with improvements in functional capacity, mobility, and self-reported physical ability in patients with KOA, with some evidence supporting a beneficial effect on gait; however, the certainty of evidence remains low to moderate due to heterogeneity among studies and limited sample sizes. These findings should be interpreted in light of the methodological limitations identified across the included trials.

## 1. Introduction

Knee osteoarthritis (KOA) is the most prevalent musculoskeletal disorder worldwide, affecting hundreds of millions of people. According to global estimates, approximately 374 million people were living with KOA in 2021, with over 30 million new cases each year and a substantial burden of more than 12 million disability-adjusted life years (DALYs). Females over 50 years of age and populations from regions with higher socio-demographic indices show increased risk. The disease affects adults and the elderly population, with an age-related increase in prevalence, leading to pain, functional limitations, and reduced quality of life (QoL) [1]. Impairments in walking ability and functional performance are the most disabling consequences for these patients, which is also demonstrated by gait analysis studies highlighting reduced physical performance in individuals with KOA [2,3].

Exercise-based rehabilitation is strongly recommended as a component of conservative management of KOA in international clinical guidelines, including those developed by the Osteoarthritis Research Society International (OARSI) [3]. However, land-based exercise programs may be poorly tolerated by some patients due to pain and functional limitations. In this context, the potential benefit of hydrotherapy is supported by biomechanical and physiological mechanisms, including reduced joint loading through buoyancy, increased muscular engagement due to water resistance, and enhanced proprioceptive feedback and circulation mediated by hydrostatic pressure, enabling safe and effective specific exercises for functional performance and gait [4].

Given that KOA directly affects walking ability and daily functional activities, the effects of rehabilitation interventions should be assessed using objective, validated outcome measures. Functional performance is commonly assessed through standardized tests, while computerized gait analysis allows detailed assessment of temporospatial, kinetic, kinematic, and neuromuscular parameters, providing valuable insights into mobility and movement strategies in patients with KOA [5]. Additionally, patient-reported outcome measures are widely used to evaluate pain, physical function, and health-related QoL. Current evidence indicates that systematic reviews on hydrotherapy in KOA [6] have primarily focused on pain reduction, self-reported physical function, and QoL [7]. Fewer studies have addressed balance, mobility, and postural control, while data on functional performance and walking-related outcomes remain limited and inconsistent [8]. Although several reviews suggest that hydrotherapy is comparable to land-based exercise in alleviating pain and improving physical function and QoL in patients with KOA in the short and long term, these findings are not entirely consistent. Variability in reported effects may be attributed to heterogeneity in study population, intervention protocols, and outcome measures, particularly in functional performance and gait-related outcomes. Compared with no intervention, hydrotherapy may improve daily activities and participation; however, its effects remain uncertain. While there is an association between better adherence, patient satisfaction, and a favorable safety profile, particularly for those with severe pain or a low tolerance for axial-loading exercises, it is unclear whether these advantages lead to better functional outcomes.

Although a growing number of studies have examined aquatic exercise in KOA, previous systematic reviews have primarily focused on patient-reported outcomes, including pain, stiffness, and perceived physical function. In contrast, less attention has been paid to objective measures of functional performance and gait-related parameters—the evidence being limited and fragmented—even though these are critical for maintaining mobility, independence, and overall daily functioning in this population. Recent RCTs have explored additional dimensions of walking ability—such as preferred walking speed, spatiotemporal gait variables, and aspects of neuromuscular control—that have not been synthesized across existing reviews. Therefore, this systematic review addresses this gap by offering a focused synthesis of objective functional performance and gait-related outcomes in patients with KOA, thereby extending the current evidence beyond previous meta-analyses.

### Objective of the Review

The aim of this systematic review was to evaluate the effect of hydrotherapy compared with land-based exercises, education, usual care, conventional treatment, or no intervention on functional performance and gait-related outcomes (primary outcome) and patient-reported outcomes (secondary outcomes) in patients with KOA.

## 2. Materials and Methods

The methodology of this systematic review followed the Preferred Reporting Items for Systematic Reviews and Meta-Analyses (PRISMA) guidelines [9], and the PRISMA checklist is provided in the Supplementary Materials (Table S1). It was designed to ensure adherence to standards of transparency and uniformity in data reporting and was prospectively registered in the International Prospective Register of Systematic Reviews (PROSPERO) [10] with registration number CRD420261297250. Minor refinements to the search strategy, such as the inclusion of additional synonyms, were performed to improve sensitivity.

### 2.1. Search Strategy

For this systematic review, an extensive search of articles in six databases (PubMed, Web of Science, Cochrane, PEDro, SpringerLink Journal, and ScienceDirect) was performed. Furthermore, Google Scholar was used as a supplementary source to identify additional relevant studies. To reduce selection bias, only the first 200 results sorted by relevance were screened. The search included studies published between January 2016 and February 2026. Only studies conducted in human participants and published in English were considered eligible.

The literature search followed the PICO (population, intervention, comparator, and outcome) framework.

The population of interest includes adults aged 40 and older diagnosed with KOA, according to the American College of Rheumatology criteria, with or without radiographic severity grading (Kellgren–Lawrence [3]).

The intervention consisted of hydrotherapy-based rehabilitation programs, including structured aquatic exercises (stretching, strength, balance, resistance training, free or treadmill walking, or dance-based exercises) performed in a therapeutic pool.

Comparators include land-based rehabilitation programs (e.g., exercise therapy, physiotherapy, and mud application), usual care, lifestyle-based interventions, regular treatment, educational programs, or no intervention.

The primary outcomes of interest were functional performance and gait-related parameters. Secondary outcomes included patient-reported measures of pain, physical function, and health-related quality of life. The review considered both functional performance tests and gait parameters as predefined outcomes. All of these variables were searched and extracted with equal methodological rigor according to the study protocol, following the results of standardized clinical tests, gait-related reported parameters [11,12,13,14,15,16,17,18], clinical outcome measures [19], and patient-reported functional scores [20,21,22,23,24,25,26].

The search strategy involved combining the following terms: “knee osteoarthritis”, “knee OA”, “gonarthrosis”, “hydrotherapy”, “aquatic exercise”, “aquatic therapy”, “water-based exercise”, “pool exercise”,” hydro-kinesiotherapy”, “gait”, “walking”, and “functional performance”. Boolean operators (“AND” and” OR”) were used to combine search terms. The search expression was adapted for each database as appropriate (Table 1).

Google Scholar was searched as a supplementary source. Due to the large number of results and the lack of advanced filtering options, only the first 200 results sorted by relevance were screened. This approach was adopted to maintain feasibility and reproducibility while minimizing selection bias, and all records were assessed using the predefined inclusion and exclusion criteria. The search was conducted by two evaluators who worked independently, with disagreements resolved by a third assessor. All references (479 in total) were imported into Zotero reference management software 9.0.3, where the records were archived and organized, and the duplicates were removed [27].

### 2.2. Study Selection

The selection of papers involved screening the titles and abstracts of the articles to determine eligibility based on the established inclusion and exclusion criteria and the PICO framework. Afterwards, the remaining articles were read in full to make the final selection. Inter-rater agreement statistics (e.g., Cohen’s kappa) between the two reviewers involved in this process were not calculated; however, disagreements during screening were minimal, and they were resolved through discussion with the third reviewer, who was consulted when necessary.

Inclusion criteria included the following:

- Randomized controlled trials (RCTs);
- Original studies involving patients with KOA over 40 years;
- KOA being clinically diagnosed using the American College of Rheumatology (ACR) criteria, without mandatory radiographic confirmation [28];
- Radiographic severity being assessed using the Kellgren–Laurence classification when reported [29];
- Studies comparing hydrotherapy with other rehabilitation interventions, usual care, educational programs, or no interventions;
- Studies assessing at least one standardized functional performance measure or one gait-related outcome (temporospatial, kinetic, or kinematic) before and after rehabilitation through hydrotherapy;
- English-language studies;
- Studies published between 2016 and 2026.

Exclusion criteria included the following:

- Studies involving children, adolescents, or adults under 40 years of age;
- Studies involving patients who underwent orthopedic surgery intervention on the lower limb or suffered from neurological diseases (central or peripheral);
- Studies that did not evaluate hydrotherapy as a rehabilitation intervention;
- Studies on non-human subjects.

### 2.3. Data Extraction

Two independent reviewers extracted data from the selected articles; any discrepancies were resolved through discussion until agreement was reached. Tables were created to include information such as the study’s authors, year of publication, number and characteristics of the population in the intervention and control groups, type and protocol of hydrotherapy as a rehabilitation treatment, the nature of the intervention in the control group, outcomes of interest (functional performance, gait-related parameters, and patient-reported outcomes), the methods and devices used, and the main findings. Data extraction was conducted using a standardized, predefined form that captured all of this information. Before the full data extraction, the form was tested on a small number of studies to ensure clarity and consistency.

### 2.4. Clinical and Functional Outcomes

#### 2.4.1. Functional Performance Tests

Functional performance was assessed using several standardized tests. The 6 min walk test (6MWT) evaluates walking endurance and submaximal functional capacity [11,12], while the 10 m walk test (10MWT) measures gait speed and functional mobility [13]. The UKK 2 km assesses aerobic capacity and walking endurance [14]. Functional mobility was evaluated using the Timed Up and Go (TUG) test [15] and the sit-to-stand (STS) test, the latter of which reflects lower-limb strength and performance [16]. The 5STS offers a practical, simple method for evaluating lower-limb performance in older adults and serves as an alternative assessment for identifying community-dwelling older adults with reduced gait speed when direct gait evaluation is not feasible. The study further suggests specific cutoff values for 5STS based on gait speeds below 0.8 m/s for women and below 1.0 m/s for men [17]. Walking speed and mobility were further assessed with the 40 m fast-paced walk test (FPWT-40). Stair negotiation ability was evaluated using the stair climb test (SCT) and stair test (ST), which measure functional mobility during stair ascent and/or descent [18]. The comprehensive descriptions of each test are provided in the Supplementary Materials (Table S2).

#### 2.4.2. Gait-Related Outcome

Gait-related outcomes were predefined as primary outcomes and were included when reported in the eligible studies. These comprised spatiotemporal parameters (step length, stride length, cadence, walking speed, and gait cycle phases), kinematic variables (knee flexion–extension angles and range of motion), and kinetic measures (knee adduction moment [KAM], KAM impulse, and ground reaction forces [GRF]). Additional gait-related variables, such as symmetry, variability, posture, and muscle activation during gait, were considered exploratory outcomes due to heterogeneous reporting across studies. All gait data were extracted as reported in the individual studies, irrespective of the measurement instrument used.

#### 2.4.3. Clinical Outcome Measures

In KOA, joint function can be affected by factors such as knee flexor and extensor muscle strength, joint range of motion (ROM), and circumferences of the lower limb (thigh, knee, and calf). These parameters, along with local pain, influence functional capacity and are important in evaluating knee function [19].

#### 2.4.4. Patient-Reported Functional Outcomes

In KOA research, the patient-reported functional outcomes are assessed using standardized tools, which include pain scales such as the Visual Analog Scale (VAS) [20] and Numeric Rating Scale (NRS) [21], disease-specific questionnaires like Western Ontario and McMaster University’s Osteoarthritis Index (WOMAC) [22] and Knee Injury and Osteoarthritis Outcome Score (KOOS) [23] and outcome measures such as the Short Form-36 [23,24], EuroQol-5D [25] and health assessment questionnaire disability index (HAQ-DI) [26].

### 2.5. Quality Assessment

The methodological quality of the included studies was assessed using the revised Cochrane risk of bias tool for randomized trials (RoB2), designed for systematic reviews, which assesses five standardized domains [30]. Two reviewers independently assessed each RCT. The results were then compared, and any discrepancies were re-evaluated through discussion. In cases where consensus could not be reached, the third reviewer was consulted. The evaluation was conducted at the outcome level, focusing specifically on functional performance outcomes and gait-related parameters. In addition, the overall certainty of the evidence was determined using the GRADE (Grading of Recommendations Assessment, Development and Evaluation) approach [31]. All included studies were RCTs, corresponding to Level 1 evidence according to the Oxford Center for Evidence-Based Medicine; therefore, additional classifications were not considered necessary.

### 2.6. Statistical Analysis

The analysis was mainly descriptive. A structured meta-analysis was not conducted because of methodological heterogeneity in the interventions and in the functional and gait parameters assessed across the included studies. The number of studies measuring the same outcome was too small to support meaningful subgroup meta-analyses. A qualitative synthesis of the selected studies was conducted, focusing on population characteristics, interventions, control groups, and outcomes assessed.

## 3. Results

### 3.1. Search Results

The initial database search identified 479 records. After removing duplicate records, 389 articles remained. Screening of titles and abstracts resulted in the exclusion of 296 studies for the following reasons: studies not addressing KOA (n = 38), studies involving surgical interventions (n = 36), absence of hydrotherapy as a rehabilitation intervention (n = 72), conference abstracts (n = 12), or publications not representing original research (n = 138).

**Figure 1:**
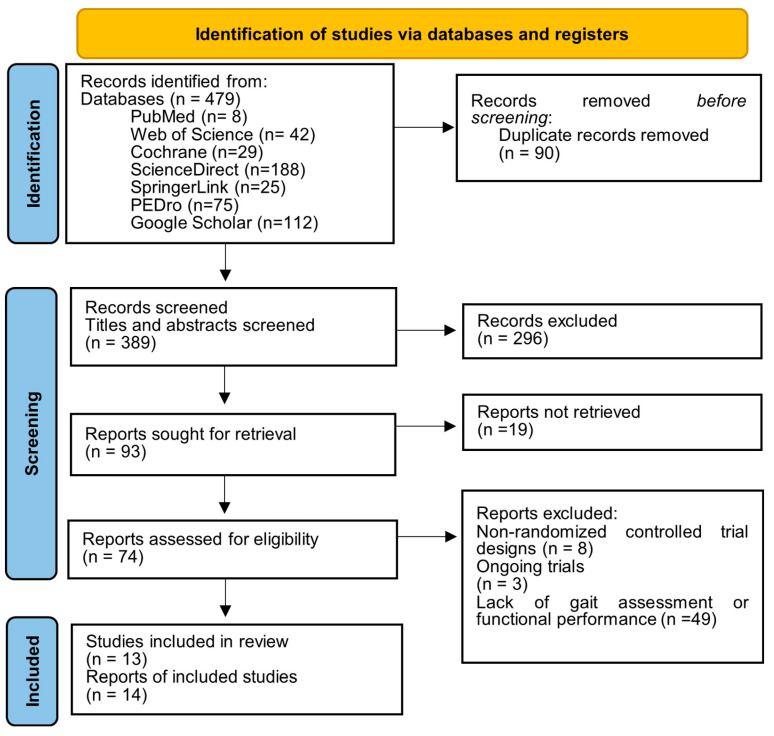
PRISMA flow diagram. Source: [9].

The full texts of 93 potentially relevant articles were evaluated for eligibility; however, 19 could not be retrieved. Consequently, 74 full-text articles were evaluated in detail in accordance with the predefined inclusion and exclusion criteria. Of these, 61 studies were excluded due to non-randomized controlled trial designs (n = 8), ongoing trials (n = 3), and the lack of gait assessment or functional performance measurement (n = 49). Two publications were identified as reporting results from the same clinical trial; therefore, data were extracted from the most complete and recent report to avoid double-counting. A total of 13 studies (reported in 14 publications) met the eligibility criteria and were included in the final qualitative synthesis (Figure 1).

The findings are reported according to the predefined primary outcomes—functional performance tests and gait-related parameters—and secondary outcomes, comprising patient-reported measures of pain, physical function, and health-related quality of life. All studies reported functional performance test results and gait-related outcomes; only two studies did not report patients’ self-reported outcomes.

### 3.2. Characteristics of Included Studies

The studies were evaluated, and the data were extracted and recorded in specific tables. The author’s names, country, number of participants in the intervention and control groups, age, body mass index (BMI), and Kellgren–Laurence (K-L) stage, when reported, were noted (Table 2).

The 13 included RCTs were conducted across diverse geographical areas, including Spain, Finland, Brazil, Thailand, Iran, Lithuania, and India, with sample sizes ranging from 16 to 290 participants. Most studies involved adults of middle age and older, though some focused on more specific populations, such as postmenopausal women and the elderly, leading to variability in baseline characteristics. A critical comparison of protocols reveals substantial heterogeneity. The duration of aqua therapy programs varied from two to seven sessions per week, with a maximum of 24 sessions delivered over periods ranging from ten days to twelve weeks. The type and intensity of aquatic interventions differed across the studies, including aquatic resistance training, walking exercises, stretching, balance training, and combined programs, reflecting a lack of standardization in exercise design. Moreover, hydrotherapy was evaluated against different control conditions, such as land-based exercises and physiotherapy, mud application, usual care or medication, education, lifestyle, or no intervention. The follow-up periods varied between 1 and 6 months. These differences in experimental and control group interventions and follow-up duration may have influenced the reported outcomes and direct comparability between studies. Another source of heterogeneity was the different reported outcome measures across studies. We grouped the studies, and only a limited number of comparable outcomes (six for the 6MWT and three for the TUG test) were reported. As a result, a quantitative synthesis was conducted.

The description of the intervention in the two groups, the time of application, the outcome, a summary of the results, and the reference number were recorded for each study (Table 3).

### 3.3. Risk of Bias

**Figure 2:**
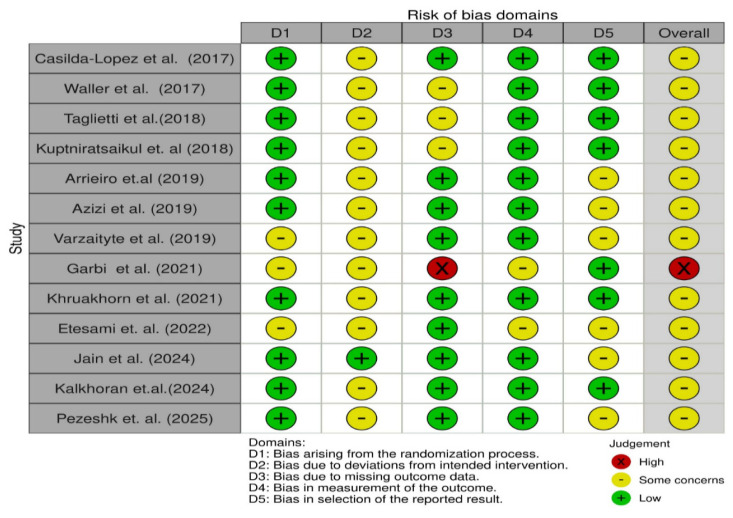
Risk of bias using RoB2 for included studies [32,33,34,35,36,37,38,39,40,41,42,43,44].

The overall risk of bias across the included studies was assessed as exhibiting “some concerns”. The RoB2 tool evaluated five domains: bias arising from the randomization process, bias due to deviations from intended interventions, bias due to missing outcome data, bias in measurement of the outcome, and bias in selection of the reported result [29,30]. Domains D1 (randomization) and D4 (outcome measurement) were judged as low risk in the majority of trials. However, several studies raised concerns in domains D2 (deviations from intended interventions) and D5 (selection of reported results), which have direct implications for the interpretation of patient-reported outcomes such as WOMAC and VAS scores, as these measures are more susceptible to performance and expectation bias. Performance-based tests (e.g., TUG and 6MWT) and gait-related parameters are inherently less influenced by subjective factors and were therefore considered more robust to these sources of bias. Only one study was rated at high risk, primarily due to concerns regarding missing outcome data (D3). These considerations were taken into account in the interpretation of the findings (Figure 2).

In rehabilitation research, blinding of participants and therapists is often difficult to achieve due to the nature of exercise-based interventions. In the included trials, both aquatic and land-based exercise programs required active participation and supervision, rendering blinding impractical and potentially increasing the risk of performance bias. In addition, patient-reported outcomes such as pain or perceived physical function may be more susceptible to expectation or performance bias, whereas performance-based tests and gait-related parameters are generally considered less influenced by subjective factors.

**Figure 3:**
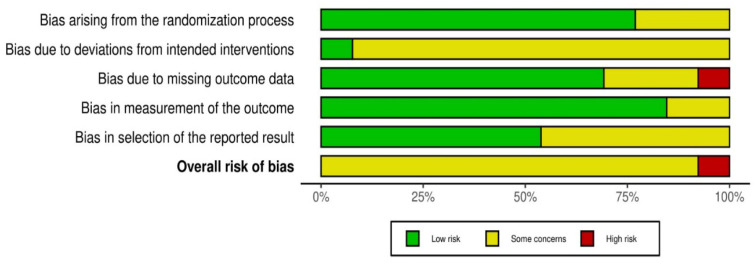
A summary plot of RoB2 for the included studies [32,33,34,35,36,37,38,39,40,41,42,43,44].

Although the included trials were generally conducted in accordance with standard RCT design principles, the methodological quality was variable. Several studies exhibited concerns related to blinding of participants and care providers—an inherent limitation of exercise-based rehabilitation research—as well as incomplete outcome data and selective reporting. These limitations should be considered when interpreting the magnitude and consistency of the reported effects. A graphical summary of the risk of bias assessment is shown in Figure 2 and Figure 3.

Given the methodological heterogeneity of the included studies, detailed study-specific data are presented in Table 3. The following sections provide a narrative synthesis of the results by outcome domain, including functional performance, gait-related parameters, and patient-reported outcomes.

### 3.4. Functional Performance Outcomes

Functional performance outcomes were evaluated in eleven of the included RCTs [32,33,34,35,36,38,39,40,41,42,43]. Improvements in the 6 min walk test (6MWT) were reported in five studies [32,35,36,39,41] with statistically significant between-group differences in favor of hydrotherapy observed in the study of Casilda et al. [32]. The Timed Up and Go (TUG) test was assessed in five studies [34,39,40,41,42], with significant improvements in the intervention group relative to controls reported in Garbi et al. and Jain et al. [39,42]. Improvements in the five times sit-to-stand (5STS) test were documented in two studies [38,40]. Varzatyte et al. noted statistical significance after mineral bath water therapy compared with the control group [38]. The stair climb test (SCT) showed significant within-group changes in the trials of Etesami et al. following 8 weeks of hydrokinetotherapy and Khruakhorn et al. after six weeks of water-based stretching exercises; the latter observed significant statistical improvement between groups [40,41]. Gait speed was clinically assessed using tools such as the UKK 2 km test and 10 m walk test in three studies [33,38,43]. In the trial of Varzaityte et al., the specific testing protocol was not described in detail in the published methodology [38]. The improvement was statistically significant in the interventional groups compared to the controls at the end of treatment, but it was not maintained at follow-up [33,38].

Overall, most studies reported improvements in functional performance following hydrotherapy intervention.

### 3.5. Gait-Related Outcomes

Gait-related parameters were evaluated using technology in two of the included RCTs [37,44], which substantially limits the strength and generalizability of any conclusions regarding the effects of hydrotherapy on walking mechanics. An additional three studies assessed walking speed using previously described clinical functional performance tests [33,38,43].

Azizi et al. investigated the effects of an 8-week aquatic exercise program in elderly men with KOA compared with a control group receiving lifestyle advice evaluating temporospatial gait parameters and balance measures [37]. Significant improvements were observed in stride length and cadence in the interventional group, while no significant changes were noted in step time or step width compared with the control group. Static and dynamic balance also improved significantly in the hydrotherapy group. Pezeshk et al. (2025) compared aqua therapy and land-based exercise over 8 weeks, finding that although both exercise modalities were beneficial, hydrotherapy produced greater biomechanical and neuromuscular improvements [44]. The hydrotherapy group showed significant improvement in knee range of motion during early stance, rectus femoris activity, and overall muscle activation, with moderate-to-large effect sizes. Compared with the land-based exercise group, hydrotherapy demonstrated larger effect sizes for knee ROM improvement, as well as greater enhancement of rectus femoris activation and reduction in hamstring activity, indicating superior optimization of muscle activation patterns during the early stance phase of gait.

While the available data suggest potential benefits in selected gait parameters—including walking speed, stride length, and cadence—the findings should be interpreted with considerable caution, given the small number of contributing studies, the heterogeneity in assessment methods, and the variability in reported outcomes.

### 3.6. Patient-Reported Functional Outcomes

Patient-reported outcomes were assessed in eleven of the included RCTs [32,33,34,35,36,37,38,39,40,42,44]. These outcomes were evaluated using validated instruments commonly applied in KOA research, including the NRS, VAS, WOMAC, KOOS, and SF-36 questionnaires.

WOMAC score was assessed in six studies, all of which showed improvements in both groups [32,34,36,39,40,42]. Statistically significant differences between interventional and control groups were noted in five studies [32,34,39,40,42]. In Casilda et al.’s study, the improvement was noted only in pain and total domains of WOMAC [32].

KOOS was assessed in two studies [33,44]. In Waller et al.’s research, no improvement was observed [33], and Pezeshk et al. in 2025 showed that the KOOS increased in both groups, with a large effect size observed in the water-exercise group, although the difference was not statistically significant [44].

The VAS scale was used in four studies [34,37,38,42] and the NRS in one study [35]. Across all trials, pain levels decreased in both groups with a statistically significant improvement in the interventional group [34,35,37,38,42].

SF-33 was evaluated in three studies, showing increased scores but no differences between groups [34,36,38].

Quality of life, as measured by the WHOQOL-100, was evaluated in one study using a specific questionnaire. QOL outcomes showed improvements in both groups at 6 weeks, with significant gains in the physical domain and in the total score in both the intervention and control groups. Additionally, the hydrotherapy group showed improvement in the environmental domain. At 6 months, only the hydrotherapy group showed sustained, significant improvements in mental and social quality-of-life scores and overall quality-of-life scores [40].

### 3.7. Certainty of Evidence (GRADE Assessment)

The certainty of evidence for the main outcomes was evaluated using the Grading of Recommendations Assessment, Development and Evaluation (GRADE) approach. The evaluation considered the domains of risk of bias, inconsistency, indirectness, imprecision, and publication bias. Downgrading decisions were operationalized as follows: for functional performance outcomes, the certainty was downgraded one level for imprecision, owing to small sample sizes in several trials and limited overlap in the specific tests used (e.g., TUG, 6MWT, and STS), resulting in moderate certainty. For gait-related outcomes, the certainty was downgraded two levels—one for serious inconsistency, reflecting substantial variability in the parameters assessed (walking speed, cadence, and stride length) and the methodological diversity of assessment instruments, and one for serious imprecision, due to the small number of contributing studies and limited sample sizes, resulting in low certainty. For patient-reported outcomes, the certainty was similarly downgraded two levels—one for serious inconsistency, attributable to the use of different validated instruments (WOMAC, KOOS, VAS, and SF-36) with distinct scoring systems and domains, and one for serious imprecision, resulting in low certainty overall. The detailed results of the GRADE assessment are presented in Table 4.

## 4. Discussion

The findings of the present review suggest that hydrotherapy may contribute to clinically meaningful improvements in functional mobility, walking capacity, and pain in patients with KOA. Locomotor performance gains were reflected by increased distance in 6MWT [32,35,36,39,41], improved TUG performance [34,39,40,41,42], enhanced walking speed [33,38,43], and better performance in SCT and STS tests. However, it is equally important to acknowledge that several trials did not demonstrate statistically significant between-group differences. Taglietti et al. (2018) reported no significant improvement in TUG performance in either the aquatic exercise or patient education group [34]. Kuptniratsaikul et al. (2018) found no significant differences between underwater treadmill and home exercise programs for the 6MWT improvement at four weeks [35]. Similarly, Arrieriro et al. (2019) and Etesami et al. (2022) reported comparable improvements in both aquatic and land-based groups across multiple functional outcomes, with no statistically significant between-group differences [36,41]. These null results are clinically relevant and suggest that the benefits of hydrotherapy, while present, are not consistently superior to other rehabilitation modalities and may be partly attributable to the general effects of structured exercise participation.

Improvements in static and dynamic balance, enhanced proprioception, and optimized muscle activation during gait were reported in a subset of trials; however, the amplitude of these improvements varied across studies, reflecting differences in intervention protocols, treatment duration, and patient characteristics. Aquatic therapy demonstrated beneficial effects on pain reduction, decreased joint stiffness, functional improvement, and activities of daily living, as measured by VAS, WOMAC, and KOOS, representing the most consistent finding across the included studies. These benefits were observed immediately after the intervention and, in some studies, were maintained at follow-up for up to three months [32,34] and up to six months [40]. Casilda et al. (2017) additionally reported increased cardiorespiratory capacity, reduced post-exercise heart rate, and decreased fatigue following aquatic interventions, suggesting systemic benefits beyond joint-related outcomes [32]. Waller et al. noted that aquatic resistance training has been associated with favorable changes in body composition, including reductions in fat mass and overall improvements, which are clinically relevant, as excess adiposity is known to contribute to KOA progression through both increased mechanical joint loading and low-grade inflammatory processes [33,45].

The observation that several studies reported outcomes in aquatic therapy comparable to those achieved with patient education or matched land-based exercise warrants critical reflection [34,36]. While this may suggest that structured physical activity, irrespective of the medium, is a primary driver of functional improvement, it does not diminish the specific value of the aquatic environment. The therapeutic mechanisms of hydrotherapy are likely multifactorial: buoyancy provides unloading of joints, hydrostatic pressure reduces periarticular edema, and thermal effects contribute to analgesia and muscle relaxation—properties that enable exercise participation in patients who would otherwise not tolerate land-based programs [4].

The RCT of Varzaityte et al. (2019) further illustrated this point: balneological intervention combining mineral sodium chloride baths or peloid therapy with physiotherapy produced significantly greater improvements in walking speed, 5TST performance, knee flexion range, and lower-limb muscle strength compared with physiotherapy alone, with sustained benefits at one-month follow-up [38]. The absence of significant differences between mineral baths and mud applications may indicate a shared therapeutic mechanism related to the thermal and mechanical properties of natural factors in this context [46,47,48].

These findings are broadly consistent with those of previously published systematic reviews and meta-analyses. Ma et al. (2022) reported significant improvements in WOMAC pain, knee extensor muscle strength, and TUG performance following aquatic physical therapy compared with no intervention, with no significant differences observed in quality of life, flexibility, or most functional outcomes [7]. The meta-analysis by Lei et al. focused on the effects of hydrotherapy versus physiotherapy on pain relief, patient-reported outcomes such as WOMAC and the Short Form 12 Health Survey, and side effects. All of these outcomes showed significant improvement in the IG at 1, 14, and 8 weeks, with no additional adverse effects [48]. Xu et al. (2022), analyzing 22 RCTs, showed improved pain relief (maintained at 3 months), stiffness, and physical function compared with no exercise, yet revealed no significant differences when compared with land-based exercise programs, and also no negative outcomes [49]. More recent evidence from Valenzuela-Fuenzalida et al. (2024) confirms significant improvements in WOMAC stiffness, pain intensity VAS score, and TUG in favor of aquatic exercise, while WOMAC pain, physical function, and KOOS outcomes showed no statistically significant superiority over other therapeutic modalities [50]. Analyzing eight RCTs of patient-reported outcomes (KOOS, VAS, and WOMAC), Dong et al. (2018) conducted a meta-analysis that indicated no significant differences between aquatic exercise and land-based exercise in pain reduction, physical function, or quality of life in patients with KOA for both short- and long-term interventions [6]. In the review by Noor et al. (2023), improvements in functional performance measures—including TUG, 6MWT, STS, and SCT—were reported following aquatic therapy [8], consistent with the present findings. Unlike previous reviews, the present study specifically targeted functional performance and gait-related outcomes, incorporating recent RCTs examining neuro-muscular control, proprioception, and range of motion, thereby extending the available evidence.

Taken together, these findings involve several clinical implications for the rehabilitation of patients with KOA. The disease’s association with significant impairments in gait and functional performance, including reduced walking speed, shorter step length, and altered loading patterns [51,52,53,54], suggest that these impairments may be partially addressed through hydrotherapy-based rehabilitation. The aquatic environment offers specific clinical advantages by reducing loading while allowing safe performance of strengthening and gait-related exercises [4], thereby facilitating participation in patients with moderate to severe pain, elevated BMI, or kinesiophobia. Nevertheless, the results of this review do not demonstrate consistent superiority of hydrotherapy over land-based exercises. Studies comparing aquatic with land-based exercises in patients with KOA generally reported improvements in pain, functional performance, and walking capacity [35,36,40,41]. Jain et al. (2024) and Pezeshk et al. (2025) observed benefits in both interventions, with slightly greater functional and neuromuscular improvements following hydrotherapy [42,44]. A combined rehabilitation approach integrating early aquatic therapy with progressive land-based training may be more effective in optimizing long-term functional outcomes and adherence. These findings suggest that hydrotherapy should be considered a complementary component within a comprehensive rehabilitation program rather than an isolated intervention, with careful patient selection based on clinical characteristics and contraindications. Moreover, aquatic therapy is a cost-effective adjunct therapy for KOA, offering a favorable balance between costs and benefits, and it is a recommended option in modern disease management [55].

Several limitations of this review should be acknowledged when interpreting the findings. The included trials exhibited substantial methodological heterogeneity across multiple dimensions, including the type, intensity, frequency, and duration of aquatic interventions; water temperature; pool depth; and the degree of therapist supervision, precluding direct protocol comparison and possibly contributing meaningfully to the variability in reported outcomes. Sample sizes across the included studies ranged from 16 to 290 participants, and the majority of trials enrolled fewer than 60 participants, which raises concerns regarding statistical power and the precision of effect estimates. Several studies did not report radiographic disease severity according to the Kellgren–Lawrence classification [29], limiting the ability to assess whether treatment responses differed across disease stages. A subset of trials did not provide detailed baseline demographic characteristics, further constraining the interpretation of subgroup-specific findings.

The absence of participant and therapist blinding—an inherent constraint of exercise-based rehabilitation research—represents an additional source of potential performance and detection bias, particularly for patient-reported outcomes such as pain intensity and perceived functional capacity, which are more susceptible to the expectation effect than objective performance-based tests. Furthermore, only a limited number of studies assessed gait-related parameters using biomechanical instrumentation; the remainder relied on clinical walking tests, which, while validated and widely used, do not capture the full complexity of gait mechanics in individuals with KOA. The heterogeneity of comparator interventions—ranging from no treatment and usual care to structured land-based rehabilitation programs—introduces further variability that limits the interpretability of between-group effect sizes across studies.

A further limitation of this review is the inability to conclude sex-based differences in response to hydrotherapy. Several included trials enrolled exclusively female populations, predominantly postmenopausal women [32,33,36,41,43,44], while mixed-sex trials did not consistently report sex-stratified outcome data [34,35,38,39,40,42]. Given the higher prevalence of KOA in women, documented sex differences in pain processing and neuromuscular function, and the potential for hormonal factors to modulate responses to aquatic exercise, future research should prioritize adequately powered trials with pre-specified subgroup analyses by sex.

The included trials were conducted across seven countries representing diverse geographical, cultural, and healthcare contexts. While this geographical breadth enhances the generalizability of the review’s findings, it also introduces potential sources of heterogeneity that are difficult to disentangle from other methodological sources of variability. Differences in population-level characteristics—including baseline BMI, radiographic disease severity, habitual physical activity levels, and access to rehabilitation services—may have differentially influenced treatment responses across study settings. Direct cross-cultural comparisons of treatment efficacy were not feasible within the current design. Future systematic reviews and primary trials should consider cultural and population-level factors as pre-specified moderators of treatment response in aquatic rehabilitation for KOA.

Outcome heterogeneity represents a major methodological limitation of this review. Across the included trials, functional performance was assessed using a wide variety of instruments—including the 6MWT, TUG, 5STS, 2KK, and 10MWT—while gait-related parameters encompassed temporospatial variables, kinematic and kinetic measures, and, when available, neuromuscular indices assessed using markedly different methodologies. Patient-reported outcomes similarly varied, with studies employing WOMAC, KOOS, VAS, NRS, and SF-36, each capturing overlapping yet distinct constructs. This diversity of measurement approaches prevented the pooling of data across studies and limited the direct comparability of results. To improve interpretive clarity, outcomes in this review were organized into three predefined domains—functional performance, gait-related parameters, and patient-reported functional outcomes—consistent with the structure of the GRADE assessment (Table 4). The absence of a universally adopted core outcome set for aquatic rehabilitation in KOA is a recognized gap in the field; adoption of standardized outcome measures, such as those recommended by OARSI [18], would substantially facilitate evidence synthesis in future systematic reviews and meta-analyses.

This review has several methodological strengths that should be acknowledged alongside its limitations. The restriction to RCTs reduces the risks of confounding inherent to observational designs and focuses on a single joint condition—knee osteoarthritis—which reduces clinical heterogeneity relative to reviews examining mixed osteoarthritis populations, allowing for a more condition-specific interpretation of findings. The systematic search strategy across seven databases, combined with independent dual-reviewer screening, data extraction, and risk of bias assessment using the RoB2 tool, adhered to PRISMA guidelines and enhanced the reproducibility of the review process. However, these procedural strengths must be interpreted in the context of the moderate-to-low certainty of evidence, as determined by GRADE, which limits the strength of any recommendations that can be derived from these findings.

Future research should prioritize standardized aquatic exercise protocols defining optimal frequency, intensity, duration, water temperature, and type of aquatic intervention for patients with KOA. Well-designed RCTs with larger sample sizes and longer follow-up periods and instrumented gait analysis are needed to evaluate the long-term effectiveness and sustainability of aquatic therapy outcomes. Further research could explore combined aquatic and land-based rehabilitation, and the use of three-arm designs, including a non-exercise control to disentangle medium-specific from general exercise effects.

## 5. Conclusions

This systematic review suggests that hydrotherapy improves functional performance, gait-related parameters, and patient-reported outcomes in individuals with KOA. Positive effects were most consistently observed for walking capacity, sit-to-stand performance, pain and self-reported physical function. However, the certainty of evidence remains low to moderate, as determined by the GRADE assessments, due to heterogeneity in intervention protocols and outcome measures, small sample sizes, and the limited number of trials assessing functional performance and gait-related methods and outcome measures. While hydrotherapy has demonstrated effects comparable to land-based exercise in several studies, current evidence does not consistently support its superiority. Nevertheless, it appears to represent a feasible and clinically relevant rehabilitation option for patients with KOA. Further high-quality studies using standardized gait analysis protocols are needed to clarify the long-term effects of hydrotherapy on gait biomechanics and functional rehabilitation for KOA patients.

## Figures and Tables

**Table 1:** Database search strategy.

Databases	Controlled Vocabulary (MeSH Where Applicable)	Search Filters Applied	Number of Results
PubMed, National Institutes of Health (NIH)	(“Osteoarthritis, Knee”[MeSH] OR “knee osteoarthritis” OR “knee OA” OR gonarthrosis) AND (“Hydrotherapy”[MeSH] OR hydrotherapy OR “aquatic exercise” OR “water-based exercise” OR “pool exercise” OR hydrokinesiotherapy) AND (“Gait”[MeSH] OR gait OR walking OR “functional performance”)	Publication period: 2016–2026 Species: humans Article: all types Language: English	8
Web of Science	(“knee osteoarthritis” OR “kneeOA” or “gonarthrosis”) AND (“hydrotherapy” OR “aquatic exercise” OR “water-based exercise” OR “pool exercise” OR” hidrokinesio-therapy”) AND (“gait” OR “walking” OR “functional performance”)	Publication date: 2016–2026 Document type: all Language: English	42
Cochrane Library	Title Abstract Keywords (“knee osteoarthritis” OR “kneeOA” or “gonartrosis”) AND (“hydrotherapy” OR “aquatic exercise” OR “water-based exercise” OR “pool exercise” OR” hidrokinesio-therapy”) AND (“gait” OR “walking” OR “functional performance”)	Publication date: 2016–2026 Publication type: trials	29
PEDro (Physiotherapy Evidence Database)	Abstract and title: “knee osteoarthritis”, “knee OA”, “gonartrosis” “aquatic therapy”, “water-based exercises”, “pool exercises”, “hidrokinesio-therapy”, “gait”, “walking”, “functional performance”.	Publication date: 2016–2026 Therapy: hydrotherapy, balneotherapy Body part: lower leg of the knee Subdiscipline: musculoskeletal Language: English	75
ScienceDirect	(“knee osteoarthritis” OR “kneeOA” or “gonartrosis”) AND (“hydrotherapy” OR “aquatic exercise” OR “water-based exercise”) AND (“gait” OR “walking” OR “functional performance”) (“knee osteoarthritis” OR “kneeOA” or “gonartrosis”) AND (“pool exercise” OR” hidrokinesio-therapy”) AND (“gait” OR “walking” OR “functional performance”)	Publication date: 2016–2026 Article type: all Language: English	188
SpringerLink	Search by keyword (“knee osteoarthritis” OR “knee OA” OR gonarthrosis) AND (“hydrotherapy” OR “aquatic exercise” OR “water-based exercise” OR “pool exercise” OR “hydrokinesiotherapy”) AND (“gait” OR “walking” OR “functional performance”)	Publication type: article Publication date: 2016–2026 Language: English	25
Google Scholar	(“knee osteoarthritis” OR “knee OA” OR “gonarthrosis”) AND (“hydrotherapy” OR “aquatic exercise” OR “water-based exercise” OR “pool exercise” OR “hydrokinesiotherapy”) AND (“gait” OR “walking” OR “functional performance”)	Article: any type, first 200 results screened Sorted by relevance Publication date: 2016–2026 Language: English	112
Total			479

**Table 2:** Characteristics of study groups.

No.	Study	Country	RCT Type	No. P	No. IG	No CG	Age IG + SD	Age CG + SD	BMI SG + SD	BMI CG + SD	K-L
1.	Casilda-Lopez et al. (2017) [32]	Spain	Double- blinded	34	17	17	65.6 (7.1)	66 (6.3)	31.6 (2.4)	33.6 (3.0)	NR
2.	Waller et al. (2017) [33]	Finland	Single-blinded	87	43	44	63.8 (2.4)	63.9 (2.4)	26.6 (3.8)	27.1 (3.5)	I–II
3.	Taglietti et al. (2018) [34]	Brazil	Single-blinded	60	31 (28)	29 (21)	67.3 (5.9)	68.7 (6.7)	29.2 (0.8)	30.4 (0.9)	I–IV
4.	Kuptniratsaikul et al. (2018) [35]	Thailand	Single-blinded	80 (70)	40 (33)	40 (37)	62.1 (6.4)	61.7 (6.9)	28.4 (3.0)	28.9 (3.2)	NR
5.	Arrieiro et al. (2019) [36]	Brazil	Single-blinded	16	8	8	68 (6)	67(3)	28.7 (5.3)	27.5 (2.0)	NR
6.	Azizi et al. (2019) [37]	Iran	Single-blinded	32	16	16	63.5 (4.7)	65.5 (3.3)	23.7 (2.0)	25.2 (2.7)	NR
7.	Varzaityte et al. (2019) [38] *	Lithuania	Single-blinded	60	30	30	61.0 (13.4)	67.9 (8.9)	29.2 (4.6)	29.8 (4.6)	I–III
				62	30	32	61.0 (13.4)	65.0 (10.8)	29.2 (4.6)	29.3 (3.9)	I–III
8.	Garbi et al. (2021) [39]	Brazil	Unblinded	29	17 (17)	16 (12)	63	64	(NR)	(NR)	I–III
9.	Khruakhorn et al. (2021) [40]	Thailand	Single blinded	34	17	17	64.8 (7.4)	57.8 (7.7)	26.3 (2.7)	27.2 (4.3)	II–III
10.	Etesami et al. (2022) [41]	Iran	Unblinded	54	27	27	NR	NR	NR	NR	NR
11	Jain et al. (2024) [42]	India	Unblinded	290	145 (143)	145 (142)	54.3 (11.3)	55.4 (10.2)	26.1 (3.7)	27.0 (3.5)	I–II
12.	Kalkhoran et al.(2024) [43]	Iran	Single-blinded	34	17	17	65.0 (1.34)	65.1 (1.6)	28.3 (NR)	28.0 (NR)	II–III
13.	Pezeshk et al. (2025) [44] **	Iran	Single-blinded	24	12	12	NR	NR	NR	NR	II–IV
				24	12	12	NR	NR	NR	NR	II–IV

* Study includes two comparison groups: hydrotherapy vs. mud application, and physical therapy and hydrotherapy vs. mud application. ** Study includes two comparison groups: hydrotherapy/land-based therapy and hydrotherapy/control (usual treatment for KOA). No. (number), No. P (total number of participants), IG (interventional group), CG (control group), BMI (body mass index), SD (standard deviation), K-L (Kellgren–Laurence radiological stage), and NR (not reported).

**Table 3:** Summary of the included studies.

No	Study	Population	Intervention Therapy	Control Therapy	Treatment Duration	Functional Performance Test/Gait-Related Outcome	Effect Size	Statistical Significance	Patient-Reported Functional Outcome and Other Outcomes	Results	Reference
1.	Casilda-Lopez et al. (2017) Spain [32]	Women—postmenopausal and overweight	Aquatic dance-based exercise Pool—32° 45 min × 3/week	Global aquatic exercises	8 weeks FU—3 months	6MWT—(m)	1.13	*p* = 0.002	WOMAC Fatigue (VAS) Post-E HR	6MWT: ↑ IG vs. CG post; ns at FU HR post-exercise: ↓ IG vs. CG (post and FU) Fatigue: resting ↓ both (ns); post-exercise ↓ IG vs. CG WOMAC: pain and total ↓ in IG (partially maintained); no change in stiffness/function	41
2.	Waller et al. (2017) Finland [33]	Women—postmenopausal	Aquatic resistance training 3 × 30 min/week	Usual care (LTPA) Optional: light stretching/social interaction, 2 × 30 min/w	4 weeks FU 8 weeks.	UKK 2 km walking test (m/s) walking speed	0.42	*p* = 0.002	KOOS LTPA Body composition (DXA)	Walking speed and LTPA ↑ IG (LTPA not maintained at FU). ↓ BMI and ↓ fat mass in IG KOOS: no significant changes.	49
3.	Taglietti et al. (2018) Brazil [34]	Women and men	Aquatic exercises Pool—32° Depth—1.2 m 60 min × 2/week Total—16 sessions	Education—physicians/pharmacists/nurses/nutritionists/psychologists, physiotherapists/physical educators 1/week—8 sessions	8 weeks FU 12 weeks.	TUG test (s)	#	#	VAS WOMAC SF-36	TUG and depressive symptoms ↓: ns (both groups) WOMAC pain: ↓ within and between groups, favors IG (post and 12-wk FU) WOMAC function: ↓ IG (post and FU) QoL: ↑ over time in IG	43
4.	Kuptniratsaikul et al. (2018) Thailand [35]	Women and men	Underwater treadmill exercise; 30 min × 3/week	Daily QS exercise at home; 30 min/day	4 weeks	6MWT—(m)	0.08	*p* = 0.426	NRS BMI Quadriceps strength	6MWT ↑ in both groups (ns between groups) Pain ↓ and QS strength ↑ in both groups (ns) Higher satisfaction and global assessment in IG	25
5.	Arrieiro N.A. et al. (2019) Brazil [36]	Women	Underwater walking 30–55 min × 3/week 1.2 m pool	Land-based walking exercises 30–55 min × 3/week	12 weeks	6MWT—(m) ST—Time (s)	#	#	WOMAC SF-36 VO2max AT	6MWT ↑ and ST ↓ in both groups (ns between groups) SF-36 ↑ 7/8 domains (without social function), both groups (ns) WOMAC ↓ in both groups (ns) VO2max and AT ↑ in both groups (ns)	35
6.	Azizi et al. (2019) Iran [37]	Men	Aquatic exercises: pool—60 min × 3/week 1.2 m, 32 °C + acetaminophen if needed	Lifestyle recommendations + acetaminophen if needed	8 weeks	Camera: Casio FH20			VAS Romberg’s test Balance error scoring system	Step length and cadence, significant ↑ in IG Static and dynamic balance ↑ in IG Step time and step width (ns) in IG Pain (VAS) ↓ in IG	36
						Step length (cm)	0.7	*p* < 0.001			
						Width (cm)	0.48	*p* = 0.17			
						Time (s)	0.75	*p* = 0.07			
						Stride length (cm)	2.38	*p* = 0.03			
						Cadence (step/min)	3.84	*p* < 0.001			
7.	Varzaityte et al. (2019) Lithuania [38]	Women and men	Mineral sodium chloride bath, 40–46 g/L, 36–38 °C, 15 min/day, + specific physical therapy (10 days)	Specific physical therapy (10 days)	10 days 1 month	5STS test (s) and walking speed	#	*p* < 0.001	VAS SF-36 KOOS ROM Muscle strength Tigh, K, calf circumferences	Walking speed, LKF, LKE, RKF, right K circumference bilateral muscle strength ↑ (IGs vs. CG) (10 days) 5STS ↓ (IGs vs. CG) (10 days) Walking speed, right K circumference, RKF, LKF, bilateral muscle strength ↑ (IGs vs. CG) (FU) VAS ↓ (IGs vs. CG) (FU) Bath and mud groups (ns) KOOS—partial ↑ (IGs vs. CG) (FU) SF-36 (ns) IGs and CG	18
				Mud applications to the waist and leg area, 36–42 °C 20 min, +specific physical therapy (10 days)		5STS test (s) and walking speed	#	ns			
8.	Garbi et al. (2021) Brazil [39]	Women and men	Water-free walking and strengthening lower-limb muscles; 60 min/session 16 sessions	No intervention	2 months	6MWT—(m)	#	*p* = 0.001	WOMAC - Pain; - Stiffness; - Function.	TUG-Test ↓, IG vs. CG 6MWT ↑, IG and CG WOMAC, ↓ IG vs. CG	29
						TUG test (s)	#	*p* < 0.001			
9.	Khruakhorn et al. (2021) Thailand [40]	Women and men	Water-based stretching exercises lower-limb muscles; 45–60 min × 3/week Pool 30–32 °C	Land-based stretching exercises for the lower-limb muscles 45–60 min × 3/week	6 weeks FU-6 months	TUG test (s)	0.06	ns	WOMAC WHOQOL-100	TUG and 5STS ↓ in IG and CG (ns) SCT ↓ IG WOMAC (pain, function, and total) ↓ in IG and CG (ns) WOMAC stiffness ↓ in CG WHOQOL-100 ↑ in IG and CG at 6 weeks (ns) WHOQOL-100 ↑ IG (FU)	46
						5STS test (s)	0.08	ns			
						SCT test (s)	0.21	*p* < 0.001			
10.	Etesami et al. (2022) Iran [41]	Women	Water exercises by standardized protocol 60 min × 3/week	Land-based exercises by standardized protocol 60 min × 3/week	8 weeks	6MWT—(m)	#	*p* > 0.005		6MWT and CST-30 ↑, TUG, SCT, and FPW-40 ↓ in both groups (ns)	30
						FPW-40(S)	#	*p* > 0.005			
						TUG Test(s)	#	*p* > 0.005			
						SCT Test(s)	#	*p* > 0.005			
						CST-30 test (s)	#	*p* > 0.005			
11.	Jain et al. (2024) India [42]	Women and men	Aqua resistance, balance, and proprioception—pool 40–50 min, 36 °C 3 × week 24 sessions	Resistance, balance, and proprioception exercises	8 weeks	TUG test (s)	0.69	*p* < 0.001	VAS WOMAC	Lower-limb muscle strength (1RM), proprioception, FPWT-40 ↑, and TUG ↓ (IG vs. CG) VAS and WOMAC scores ↓ in both groups (IG > CG)	21
						FPW-40 (s)	1.08	*p* < 0.03			
12.	Kalkhoran et al.(2024) Iran [43]	Women	Aquatic therapy 60 min × 2/weeks Pool 34–36 °C	Usual care	8 weeks	10MWT—Gait speed (m/s) normal	1.36	*p* < 0.001		Gait speed ↑ in normal and dual task conditions in IG vs. CG K proprioception ↑ in IG vs. CG	40
						10MWT—Gait speed (m/s) dual-task condition	1.23	*p* < 0.001			
						K proprioception	1.22	*p* < 0.003			
13.	Pezeshk et al. (2025) Iran [44]	Women	Water therapy 60 min/session Total of 18 sessions Pool 32 °C	Land-based exercises	8 weeks	Dual-camera system (12 MP) (ROM) EMG (RMS, DCCR, NET)			KOOS	K flexion range in early stance ↑ in both exercise groups, with a larger effect size for hydrotherapy (ns) KOOS ↑ in both groups; larger effect size in the water exercise group (ns)	27
						ROM	#	#			
						RMS	#	#			
						DCCR	#	*p* = 0.13			
						NET	#	*p* = 0.017			
				Regular treatmentsupervised by specialists	8 weeks	ROM early stance	#	*p* = 0.01	KOOS	RMS, NET, and DCCR ↑ exercise groupsNET and RSM (only for RF, SM) ↑ in the water vs. land exercises group	
						RMS-RF	#	*p* = 0.02			
						RMS-SM	#	*p* = 0.002			
						DCCR	#	*p* = 0.017			
						NET	#	*p* = 0.001			

# (cannot be calculated or extracted), min (minutes), FU (follow-up), 6MWT (six-minute walking test), m (meters), WOMAC (Western Ontario and McMaster University’s Osteoarthritis Index), VAS (Visual Analog Scale), Post-E HR (post-exercise heart rate), HR (heart rate), ↑ (statistically significant improvement), ↓ (statistically significant decrease), IG (interventional group), vs. (versus), CG (control group), ns (no significant differences between groups), LTPA (leisure-time physical activity), UKK 2 km (UKK Institute for Health Promotion Research 2 km test), m/s (meters/seconds), KOOS (Knee Injury and OA Outcome Score), DXA (dual-energy X-ray absorptiometry), BMI (body mass index), TUG test (timed up to go test), SF-36 (Short Form-36), QoL (quality of life), QS (quadriceps stretching), NRS (Numeric Rating Scale), ST (stair test), s (seconds), VO2max (maximum oxygen consumption), AT (anaerobic threshold), °C (degrees Celsius), ROM (range of motion), LKF (left knee flexion), LKE (left knee extension), RKF (right knee flexion), K (knee), 5STS (five times sit-to-stand), IGs (intervention groups), WHOQOL-100 (self-quality assessment scale developed by the World Health Organization), SCT (stair climb test), 1RM (one-repetition maximum), FPWT-40 (40 m fast-paced walk test), CST-30 (30 s chair stand test), 10MWT (10 m walk test), EMG (electromyography), RMS (average muscle activity), DCCR (direct co-contraction rate), NET (total muscle activation), RF (rectus femoris), SM (semimembranosus).

**Table 4:** GRADE assessment.

Certainty Assessment							No. of Patients		Effect		Certainty	Importance
No. of Studies	Study Design	Risk of Bias	Inconsistency	Indirectness	Imprecision	Other Considerations	[Intervention]	[Comparison]	Relative (95% CI)	Absolute (95% CI)		
**Functional performance (assessed by 6-MWT, TUG,5-STS test, and SCT-test)**												
11	randomized trials	not serious	not serious	not serious	serious ^a^	none	0/422 (0.0%)	0/422 (0.0%)	not estimable		Moderate ^a^	critical
**Gait-related outcome (assessed by walking speed, stride length, and cadence)**												
4	randomized trials	not serious	serious ^b^	not serious	serious ^c^	none	0/75 (0.0%)	0/75 (0.0%)	not estimable		Low ^b,c^	critical
**Patient-reported functional outcome (assessed by WOMAC, KOOS, VAS, and SF-36)**												
11	randomized trials	not serious	serious ^d^	not serious	serious ^e^	none	0/406 (0.0%)	0/376 (0.0%)	not estimable		Low ^d,e^	critical

CI: confidence interval. Explanations: ^a^ The certainty of evidence was downgraded for imprecision due to the relatively small sample sizes in several of the included trials and the limited number of studies reporting comparable functional performance outcomes. This may reduce the precision of the estimated effects and limit the robustness of the overall conclusions. ^b^ Inconsistency was judged to be serious due to variability in the gait-related outcome measures across studies, including differences in parameters such as walking speed, cadence, and stride length. In addition, the intervention protocols and assessment methods varied substantially between trials, which may have contributed to heterogeneity in the reported results. ^c^ Imprecision was judged to be serious due to the relatively small number of studies and limited sample sizes contributing to this outcome. These factors reduce the precision of the estimated effects and limit the robustness of the conclusions regarding gait-related parameters. ^d^ Inconsistency was judged to be serious due to the use of different patient-reported outcome measures across the included studies, including WOMAC, KOOS, and other validated questionnaires assessing functional status, which assess functional status using different scoring systems and domains. This methodological variability may have contributed to heterogeneity in the reported results. ^e^ Imprecision was judged to be serious due to the relatively small sample sizes in several trials and the variability in the patient-reported functional outcome measures used across studies.

## Data Availability

The original contributions presented in this study are included in the article/Supplementary Materials. Further inquiries can be directed to the corresponding authors.

## Supplementary Materials

The following supporting information can be downloaded at https://www.mdpi.com/article/10.3390/medicina62050994/s1: Table S1: PRISMA checklist, Table S2: Functional performance tests description.

## Abbreviations

The following abbreviations are used in this manuscript:

#	Cannot be calculated or extracted
&	And
↑	Statistically significant improvement
↓	Statistically significant decrease
°C	Degrees Celsius
1RM	One-repetition maximum
10MWT	10-meter walk test
5STS	The five times sit-to-stand
6MWT	Six-minute walking test
ACR	American College of Rheumatology
AT	Anaerobic threshold
BMI	Body mass index
CG	CG
cm	Centimeter
DCCR	Direct co-contraction rate
DXA	Dual-energy X-ray absorptiometry
EMG	Electromyography
FPW-40	40 m fast-paced walk test
FU	Follow-up
GRADE	Grading of recommendations assessment, development and evaluation
GRF	Ground reaction forces
HAQ-DI	Health assessment questionnaire disability index
HR	Heart rate
IG	Interventional group
IGs	Intervention groups
KAM	Knee adduction moment
K-L	Kellgren–Laurence radiological stage
KOA	Knee osteoarthritis
KOOS	Knee injury and OA outcome score
LKE	Left knee extension
LKF	Left knee flexion
LTPA	Leisure-time physical activity
m	Meters
m/s	Meters/seconds
MeSH	Medical subject headings
min	Minute
NET	Total muscle activation
No	Number
No P	Total number of participants
NR	Not reported
NRS	Numeric rating scale
ns	No significant
OARSI	Osteoarthritis Research Society International
PICO	Population, intervention, comparator, and outcome
Post-E HR	Post-exercise heart rate
PRISMA	Preferred reporting items for systematic reviews and meta-analyses
PROSPERO	International Prospective Register of Systematic Reviews
QS	Quadriceps stretching
QoL	Quality of life
RCT	Randomized controlled trial
RF	Rectus femoris
RMS	Average muscle activity
RKF	Right knee flexion
RoB2	Risk of bias tool for randomized trials
ROM	Range of motion
s	Second
SCT	The stair climb test
SCT-30	30 s chair stand test
SD	Standard deviation
SF-36	Short Form-36
SM	Semimembranosus
ST	Stair test
TUG Test	Timed Up and Go test
UKK 2 km	UKK Institute for Health Promotion Research 2km test
VAS	Visual Analogue Scale
VO2max	Maximum oxygen consumption
vs.	Versus
WHOQOL-100	Self-quality assessment scale developed by the World Health Organization
WOMAC	Western Ontario and McMaster University’s Osteoarthritis Index
